# Haemolytic uremic syndrome as a cause of chronic kidney disease stage 5 in children is in retreat: results from the Polish Registry of Kidney Replacement Therapy in children (2000–2023)

**DOI:** 10.1007/s00467-024-06584-2

**Published:** 2024-11-16

**Authors:** Ilona Zagożdżon, Maria Szczepańska, Jacek Rubik, Katarzyna Zachwieja, Anna Musielak, Monika Bratkowska, Irena Makulska, Katarzyna Niwińska, Beata Leszczyńska, Beata Bieniaś, Katarzyna Taranta-Janusz, Hanna Adamczyk-Kipigroch, Aleksandra Żurowska

**Affiliations:** 1 Department of Pediatrics, Nephrology and Hypertension, Medical University of Gdansk, Debinki 7, 80-211 Gdansk, Poland; 2 Department and Clinic of Pediatrics, Nephrology and Endocrinology, Medical University of Silesia, Zabrze, Poland; 3 Department of Nephrology and Kidney Transplantation, The Childrens Memorial Health Institute, Warsaw, Poland; 4 Department of Pediatric Nephrology and Hypertension, Jagiellonian University Medical College, Krakow, Poland; 5 Department of Pediatric Cardiology and Nephrology, Poznan University of Medical Sciences, Poznan, Poland; 6 Department of Pediatrics, Immunology and Nephrology, Polish Mother’s Memorial Hospital – Research Institute in Lodz, Lodz, Poland; 7 Department of Pediatric Nephrology, Wroclaw Medical University, Wroclaw, Poland; 8 Department of Pediatrics, Nephrology and Dialysis, Pomeranian Medical University, Szczecin, Poland; 9 Department of Pediatrics and Nephrology, Medical University of Warsaw, Warsaw, Poland; 10 Department of Pediatric Nephrology, Medical University of Lublin, Lublin, Poland; 11 Department of Pediatrics and Nephrology, Medical University of Bialystok, Bialystok, Poland; 12 Department of Nephrology, Children Hospital, Torun, Poland

**Keywords:** Haemolytic uremic syndrome, Chronic kidney disease, Epidemiology, Survival, Children

## Abstract

**Background:**

Haemolytic uremic syndrome (HUS) is a life-threatening disease with a historically poor prognosis in children receiving maintenance kidney replacement therapy (KRT). This study aimed to analyse the incidence and outcome of chronic kidney disease stage 5 (CKD5) due to *Escherichia coli*-HUS (STEC-HUS) and complement-mediated HUS (CM-HUS) in children, compared with controls with non-HUS CKD5 over the last 24 years.

**Methods:**

The study included 1488 children undergoing KRT in Poland between 2000 and 2023. Thirty-nine patients with CM-HUS and 18 with STEC-HUS were identified and analysed for incidence, KRT modality and survival.

**Results:**

The incidence rate of CKD5 was 0.09 cases/million age-related population (marp) for STEC-HUS and 0.23/marp for CM-HUS, while no new cases have been observed in recent years. CKD5 due to CM-HUS developed significantly earlier from initial HUS manifestation than in STEC-HUS (median 0.2 vs. 9.8 years). CM-HUS was associated with younger age at initiation of KRT compared to STEC-HUS and non-HUS controls (median 6.0 years vs. 10.9 and 10.9 years), with higher risk of death (Hazard Ratio 1.92, 95% confidence interval 0.9–4.13) and worse 5-year kidney graft survival at 77%, 93% and 90%, respectively (*p* < 0.001).

**Conclusions:**

In recent years, both CM-HUS and STEC-HUS have become increasingly rare causes of CKD5 in children. CKD5 due to CM-HUS in the eculizumab era and due to STEC-HUS after improving supportive treatment is exceptional. Children on KRT due to STEC-HUS had a significantly better survival, shorter waiting time for kidney transplantation and better kidney graft survival compared to the CM-HUS group.

**Graphical Abstract:**

**Figure:**
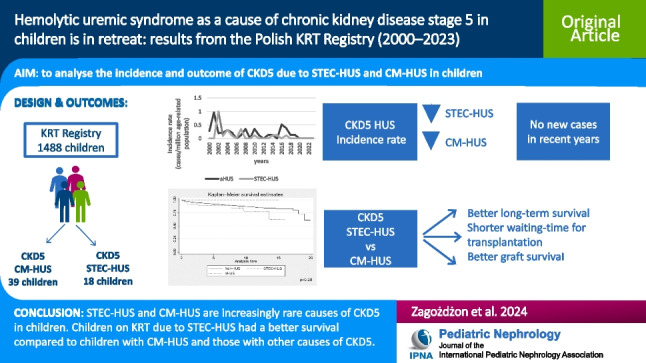
A higher resolution version of the Graphical abstract is available as Supplementary information.

**Supplementary Information:**

The online version contains supplementary material available at 10.1007/s00467-024-06584-2.

## Introduction

Haemolytic uremic syndrome (HUS) is a leading cause of thrombotic microangiopathy in childhood, characterised by haemolytic anaemia, thrombocytopenia, and acute kidney injury (AKI). The kidneys are the organs at highest risk of irreversible damage during the disease, though in 30% of cases, involvement of other organs such as the central nervous system, heart, lungs, and gastrointestinal tract can be observed, which worsens overall prognosis [1–4]. HUS in childhood is in the vast majority of cases (approximately 90%) caused by Shiga toxin-producing *Escherichia coli (STEC)* infections [5]. The most common STEC serotype causing HUS is O157:H7, but other serotypes of Shiga toxin-producing *E.coli* have been increasingly found in recent decades, including STEC O104:H4, O26 and O80 [1]. In rare cases, HUS is caused by other infectious agents such as *Streptococcus pneumoniae*, AH1N1 influenza virus, or coronavirus COVID-19. Over 10 percent of cases in children are complement-mediated HUS (CM-HUS), formerly named atypical HUS (aHUS), associated with uncontrolled activation of the alternative pathway of the complement system [6, 7]. CM-HUS can be sporadic or familial; it is usually recurrent. In both paediatric and adult populations, about half of the cases are genetically determined or associated with uncontrolled activation of the alternative pathway of the complement system [6, 7]. In a small proportion of cases in the paediatric population, HUS is diagnosed secondary to other chronic diseases such as autoimmune diseases, during radio- or chemotherapy, after bone marrow or vascular organ transplantation or as a result of some medications [8–10]. HUS is a rare disease in children, and the incidence varies considerably depending on the data source and geographical region, but collectively for STEC-HUS and CM-HUS, it is estimated at an average of 6.3–14.2/million paediatric population/year [7, 11]. The incidence of CM-HUS in children is relatively constant at 0.25–2/million/year [6, 7, 12, 13]. The clinical course and prognosis of the disease depend on the underlying cause. Seventy percent of children with STEC-HUS, assessed 5 years after an episode of HUS, fully recover. The remainder show hypertension (9%), proteinuria (18%), reduced glomerular filtration rate (7–14%), and/or neurological symptoms (4%) [14, 15]. After the acute period of the disease, 7.3% of children require chronic dialysis therapy, but in some of them, an improvement in kidney function is observed over the next few months, allowing kidney replacement therapy (KRT) to be abandoned or temporarily discontinued [16, 17]. In long-term follow-up, 1.4–4% of children are diagnosed with chronic kidney disease stage 5 (CKD5) after STEC-HUS [7, 14, 15]. In recent years, the recommendation of the intensive symptomatic treatment in the acute phase of STEC-HUS to prevent multi-organ damage has been implemented [18]. According to this approach, early fluid expansion, even in oligoanuric children, reduces the risk of thrombus formation and ischaemic damage to the kidneys and central nervous system and improves short- and long-term outcomes [19–21]. The clinical course and prognosis of CM-HUS have dramatically improved since the introduction of eculizumab—a monoclonal, humanised anti-C5 antibody that inhibits activation of the alternative complement pathway. Historically, treatment of aHUS included infusions of fresh frozen plasma or plasma exchange, but the results were not satisfactory—mortality in the paediatric population, at the first episode of the disease, was 6.7–15%, and 16–50% of patients subsequently developed CKD5 [7, 12, 22–24]. The recurrence rate was approximately 40%, and the recurrence rate in the first year after kidney transplantation was 50–90%, with an 80–90% risk of kidney graft loss [12, 24]. The introduction of eculizumab in the acute phase of the disease prevents or reduces the risk of irreversible kidney damage. For kidney transplant patients, the drug, when administered prophylactically, prevents relapse in the transplanted kidney [25]. To date, no data from national or supranational KRT registries have yet been published showing a change in the incidence of CKD5 due to HUS and a change in the long-term course of CM-HUS following the introduction of eculizumab and after volume expansion management for STEC-HUS. According to data from the ANZDATA registry, the overall survival of children with CKD5 and HUS was not significantly different from other diagnosis groups, although significantly shorter kidney graft survival was observed, but the summary did not distinguish between aHUS and STEC-HUS and was analysed in the pre-eculizumab period and before intensive symptomatic treatment was recommended.

The aim of the presented study was to discover the trends in the incidence and outcome of CKD5 due to HUS, in the population of children undergoing KRT in Poland in the last 24 years (2000–2023).

## Material and methods

**Fig. 1:**
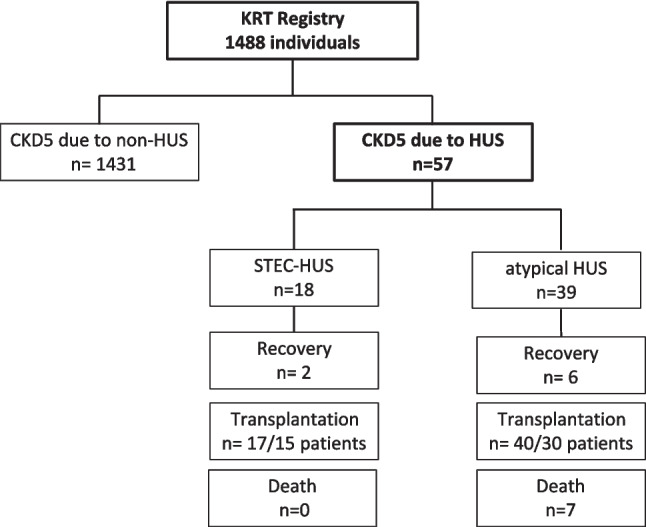
Patient selection and outcome

The prospective cohort study included 100% of the child population diagnosed with CKD5 < 18 years of age in Poland between 2000 and 2023 who had received KRT for at least 3 months (Fig. 1). The analysis of the incidence of HUS as a cause of CKD5 and the assessment of survival rates of this cohort in comparison to children with CKD5 of non-HUS origin was performed. The impact of the aetiology of HUS, choice of initial KRT, availability of anti-C5 therapy, and other factors modifying incidence and survival were analysed. Pseudo-anonymised, individual patient data were entered into the Polish Registry of Kidney Replacement Therapy in Children by the nephrologists treating them at all paediatric dialysis stations and paediatric kidney transplant centres in Poland. Information collected included demographic data, cause of kidney failure according to ESPN/ERA-EDTA codification, date of initiation and method of KRT, dates of all method changes and their types, as well as anthropometric measurements and blood pressure values. Extended clinical data including biochemical tests, pharmacotherapy, and a detailed history with detailed information on the start of nephrology care were collected for patients who had started KRT since 2008. Information on concomitant diseases, reasons for change in KRT method, reasons for kidney graft loss, and date and cause of death were also recorded. Data were updated at the end of each year of follow-up. Approval was obtained from the Bioethics Committee of the Medical University of Gdansk for the collection and processing of registry data for scientific purposes (No NKBBN/280/2018); patients and their caregivers signed an informed consent to participate in the study.

The data of patients diagnosed with CKD5 due to HUS were compared with a control group of children with non-HUS causes of CKD5 and further between the two main aetiologies: STEC-HUS and CM-HUS. Due to the use of the name aHUS in the source data of the KRT registry, as well as its use in the cited literature, the nomenclature aHUS was used in the statistical studies and discussion. Survival was assessed in each patient group, where the endpoint was death or improvement of kidney function, defined as withdrawal from dialysis therapy. The end of the follow-up observation was after attaining the age of majority or loss to follow-up. The time from the start of nephrology care (defined as first visit to a nephrologist for non-HUS patients and first manifestation of the disease for HUS patients) to the start of KRT, the time from the start of KRT to transplantation, change of dialysis modality, and kidney graft survival were also assessed, where the endpoint was the date of initiation of dialysis, after loss of graft function or the date of the next kidney transplantation. Based on the structure of the paediatric population in Poland during the analysed period, standardised incidence rates for CKD5 due to aHUS and STEC-HUS were calculated for the population of children in Poland in the years 2000–2023.

### Study population

In the period 1.01.2000–31.12.2023, 1488 children initiating KRT were reported to the Polish Registry of Kidney Replacement Therapy in Children. Of these, 59 (4%) were identified as CKD5 caused by HUS. Two of the 59 subjects with HUS secondary to systemic disease (systemic lupus in one and haemophagocytic syndrome following bone marrow transplantation in the second) were excluded from further analysis as HUS cohort (Fig. 1). Thirty-nine (3%) subjects with CKD5 as a consequence of atypical HUS and 18 (1.3%) subjects with CKD5 due to STEC-HUS were identified; the remaining 1431 were children with primary kidney disease diagnoses other than HUS (non-HUS control group). The baseline characteristics of the study groups and the control groups are detailed in Table 1. In the entire study population, the majority were boys, with no significant differences in gender distribution between groups. The median age at initiation of KRT was significantly lower in the aHUS group, 6.0 years (interquartile range [IQR] 3.4–11.1 years) compared with STEC-HUS, 10.9 years (IQR 4.1–14.9 years) and non-HUS CKD5 group, 10.9 years (IQR 5.7–14.7 years).

**Table 1:** Clinical characteristics of study population

	non-HUS CKD5	CKD5 due to aHUS	CKD5 due to STEC-HUS	*p Value*
Number	1431 (96%)	39 (3%)	18 (1%)	
Gender				
Female	638 (45%)	14 (36%)	6 (33%)	0.36
Male	793 (55%)	25 (64%)	12 (67%)	
Age at start of KRT, years (IQR)	10.9 (5.7–14.7)	6.0 (3.4–11.1)	10.9 (4.1–14.9)	0.01
Family history of CKD	9.8%	2.5%	0	0.13
Time from 1st visit to KRT onset, years (IQR)	1.5 (0–6.1)	0.2 (0–0.43)	9.9 (0–13.3)	0.0002
Hypertension at start*	34%	42%	66%	0.01
KRT first method*				0.7
Peritoneal dialysis	52%	62%	72%	
Haemodialysis	39%	38%	17%	
Transplantation	9%	0	11%	
Recovery	29 (2.5%)	6 (15%)	2 (11%)	< 0.001
Kidney transplantation (recipients)	1117 (78%)	30 (79%)	15 (83%)	0.7
Donor type				0.13
LRD	174 (12%)	2 (5%)	0	
Cadaver	1293 (88%)	38 (95%)	17 (100%)	
Number of grafts/patient**				0.71
1	837	22	12	
2	261	6	1	
3	18	2	1	
4	1	0	0	
Death	140 (9)	7 (18)	0	0.09

*Hypertension at start and KRT first method provided since 2008 for 497 individuals; **1467 kidney grafts among 1117 individuals of the control group, 40 grafts among 30 individuals of the aHUS group, and 17 grafts among 15 individuals of the STEC-HUS cohort were transplanted; *IQR* interquartile range

### Statistical analysis

Continuous variables were reported as mean ± standard deviation or as median ± interquartile range and categorical variables as counts and percentages. For the comparison of continuous variables between two groups, we used Student’s *t*-test or Mann–Whitney *U*-test as appropriate and of categorical variables the Chi-square test. The standardized incidence rates for CKD5 due to aHUS and STEC-HUS were calculated by the direct standardization using the World Health Organization world standard population distribution for age-related population. Analysis of survival was performed with the Kaplan–Meier method, and the log-rank test was used to test the null hypothesis of no difference in survival between groups of patients. Cox proportional hazard logistic regression was used in multivariable analysis and KRT method was included as time-dependent variable. The hazard ratios (HR) with 95% confidence intervals (CI) for mortality or time to the event were calculated for the presence of aHUS or STEC-HUS compared with reference population without these conditions in univariate analysis and after adjustment for age, sex, and KRT method. The Kendall tau coefficient was used to test for the presence of a trend in the incidence rate data for the period 2000 to 2023. All analyses were carried out using STATA 17.0 software (StataCorp).

## Results

### Trends in the incidence

**Fig. 2:**
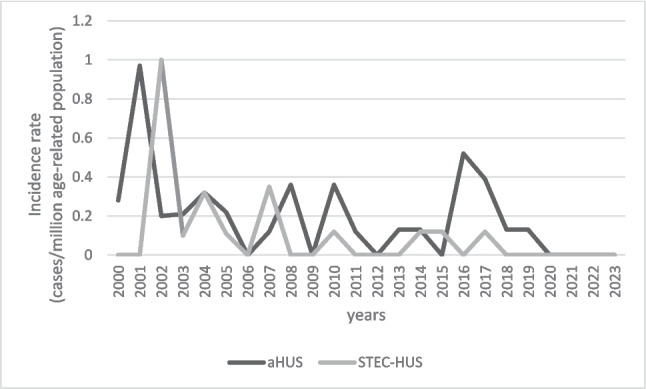
The annual incidence of CKD5 due to aHUS and STEC-HUS over the 24-year observation period; *aHUS* atypical haemolytic uremic syndrome, *STEC-HUS* Shiga toxin-producing *Escherichia coli*-haemolytic uremic syndrome

The mean age-standardized annual incidence for CKD5 due to HUS was 0.19 cases/million age-related population (marp) for aHUS and 0.09/marp for STEC-HUS. A decreasing incidence for CKD5 due to HUS was observed during the 24-year follow-up, statistically significant for aHUS (p for trend 0.03), but not significant for STEC-HUS (p for trend 0.1). No child has started KRT for STEC-HUS since 2018 and for aHUS since 2020 (Fig. 2).

**Fig. 3:**
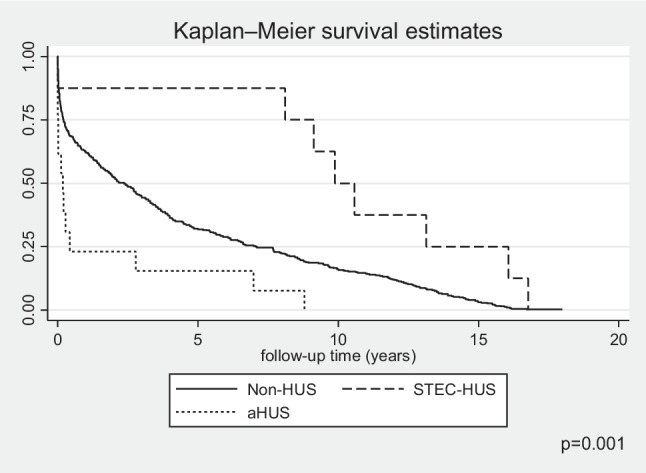
Kaplan–Meier plots for time to kidney replacement therapy initiation from onset of nephrology care; *STEC-HUS* Shiga toxin-producing *Escherichia coli-*haemolytic uremic syndrome, *aHUS* atypical haemolytic uremic syndrome, *non-HUS* other causes of chronic kidney disease stage 5

**Fig. 4:**
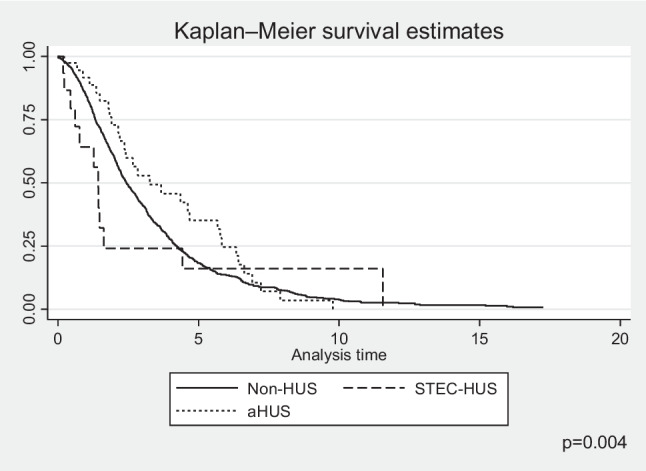
Kaplan–Meier plots for time to transplantation according to diagnosis, *STEC-HUS* Shiga toxin-producing *Escherichia coli*-haemolytic uremic syndrome, *aHUS* atypical haemolytic uremic syndrome, *non-HUS* other causes of chronic kidney disease stage 5

**Fig. 5:**
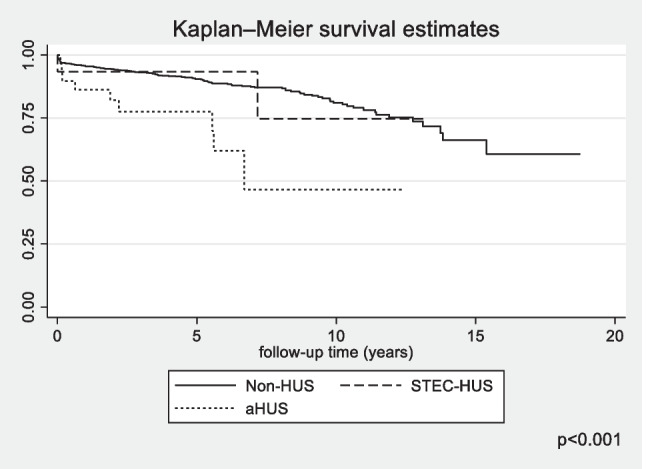
Kaplan–Meier plots for time to graft failure according to diagnosis; *STEC-HUS* Shiga toxin-producing *Escherichia coli*-haemolytic uremic syndrome, *aHUS* atypical haemolytic uremic syndrome, non-HUS other causes of chronic kidney disease stage 5

The presence of a family history of chronic kidney disease (CKD) was analysed, finding a family burden in 9.8% of the non-HUS control group, in 2.5% in the aHUS group, while there was not a single family member with CKD in the STEC-HUS group (Table 1). The time to progression of kidney disease, calculated as a median, from the start of nephrology care for non-HUS patients or first manifestation of HUS to the start of KRT, was shortest for children with aHUS, longer for the control group, and longest for STEC-HUS and represented 0.20 (IQR 0–0.43), 1.5 (IQR 0–6.1), and 9.8 (IQR 0–9.8) years, respectively (Fig. 3). Nearly 75% of aHUS patients developed CKD5 within one year of the first manifestation of the disease (Fig. 3). In 8 children with aHUS, no improvement was observed after the first episode, and maintenance KRT was initiated at the time of aHUS diagnosis. In the HUS cohort, children were significantly more likely to be hypertensive from the start of KRT (42% in aHUS, 66% in STEC-HUS) compared with the control group (34%; *p* = 0.01) (Table 1). In the majority of HUS cases, peritoneal dialysis (PD) was the first method of KRT, regardless of age; 62%, of children with aHUS, 72% following STEC-HUS; and 52% of the control group initiated KRT with PD. Haemodialysis (HD) was the initial choice for 38% of children following aHUS and 37% of the control group. For subjects with CKD5 following STEC-HUS, HD was chosen less frequently (17%). Pre-emptive kidney transplantation was performed in 11% of children with STEC-HUS and 9% in the non-HUS control group, whereas it was not performed in any patient with aHUS (Table 1). During follow-up, most children with HUS underwent kidney transplantation, which was performed in 30/38 (79%) aHUS patients and 15/18 (83%) STEC-HUS patients. One aHUS patient underwent simultaneous kidney and liver transplantation before the eculizumab availability period. The shortest waiting time for transplantation from the start of KRT was observed for STEC-HUS, with a median of 1.02 years (IQR 0.2–2.5), compared with 1.89 years (IQR 0.9–3.3) in the control group, and was significantly longer in the aHUS group, with 2.36 years (IQR 1.21–5.67; *p* = 0.004) (Fig. 4). The overwhelming majority of transplants in both the aHUS, STEC-HUS, and control groups were from deceased donors, accounting for 95%, 100%, and 88% of the respective groups. Transplantation from a living donor was not performed in any child with STEC-HUS and in only 2 children (5%) with aHUS, while in the non-HUS CKD5, it was performed in 12% of recipients. The majority of children did not require re-transplantation during the follow-up period; 75% had sustained good function of the first kidney graft, while 291 patients (25%) underwent kidney re-transplantation 2 to 4 times, including 8 patients with aHUS (27%), 2 STEC-HUS (11%), and 280 (25%) from the control group (Table 1). Median time to kidney graft loss was shortest for aHUS (2.96 years; IQR 1.7–5.6) compared with STEC-HUS (5.07 years; IQR 0.9–7.27) and the control group (3.98 years; IQR 0.8–7.28). The probability of graft loss was highest in the aHUS group (HR 3.44, 95% CI 1.74–6.83; *p* < 0.001) (Fig. 5).

### Outcome of kidney replacement therapy

#### Recovery

During the first year of dialysis therapy, 11% of STEC-HUS patients and 15% of aHUS patients, significantly more often than in the control group (2.5%; *p* < 0.001), showed an improvement in kidney function allowing withdrawal of dialysis therapy (Table 1). In two children after STEC-HUS, the improvement in kidney function persisted until the end of the observation period, i.e. until the age of 18 years. In the aHUS group, in three out of six, the improvement in function was maintained until 18 years of age; in the remaining 3, the median time to return to KRT was 2.75 years (1.12–4.64).

#### Survival

**Fig. 6:**
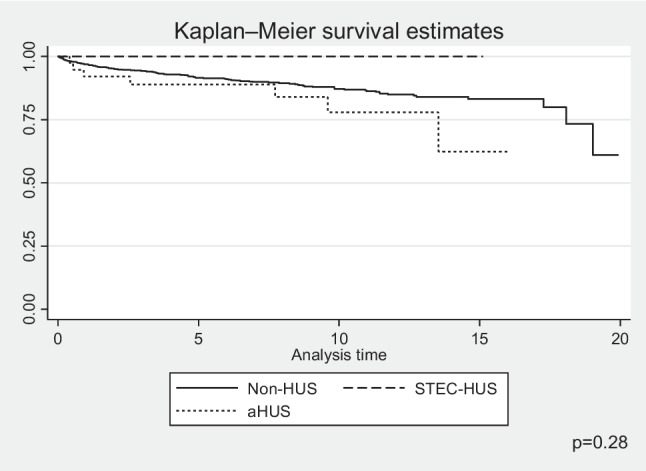
Kaplan–Meier plots for survival for STEC-HUS, aHUS and non-HUS control patients; *STEC-HUS* Shiga toxin-producing *Escherichia coli-*haemolytic uremic syndrome, *aHUS* atypical haemolytic uremic syndrome, *non-HUS* other causes of chronic kidney disease stage 5

The 1-year and 5-year survivals of patients with aHUS were 92% (95% CI 0.77–0.97) and 89% (95% CI 0.73–0.95), respectively, and was poorer than that for children with non-HUS causes of CKD5 where it reached 96% (95% CI 0.95–0.97) and 91.5% (95% CI 0.89–0.93), respectively. The differences in survival between these groups increased at 10 years follow-up: 78% (95% CI 0.55–0.9) for aHUS vs. 87% (95% CI 0.84–0.89) for non-HUS CKD5. The survival of children with CKD5 due to STEC-HUS was 100% in each analysed period, with no deaths during long term follow-up (Fig. 6). In multivariate survival analysis, independent risk factors for death were younger age (HR 0.9, 95% CI 0.87–0.93; *p* < 0.001) and aHUS diagnosis (HR 1.92, 95% CI 0.9–4.13; *p* = 0.09). The risk of death differed according to the KRT method used. In the time-dependent KRT modality analysis, the factor significantly improving survival in all diagnosis groups was kidney transplantation: HR 0.63 (95% CI 0.58–0.67; *p* < 0.001) (Table 2). There was a slightly higher risk of death for children on PD, HR 1.07 (95% CI 1.002–1.14; *p* = 0.04), but not for those on haemodialysis, HR 0.98 (95% CI 0.92–1.05; *p* = 0.75). During the 24-year follow-up, 147 deaths were reported among 1488 treated patients, including 140 deaths in the non-HUS control group, 7 in aHUS, and no deaths in the STEC-HUS patient group. The main cause of death was cardiovascular complications reported in 43 patients of the control group (40%) and in 4 in the aHUS group (57%; *p* = 0.02). Detailed causes of death in each group are included in Table 3.

**Table 2:** Unadjusted and adjusted hazard ratio for mortality risk for HUS in comparison to non-HUS CKD5

	Univariable analysis			Multivariable analysis		
	HR	*p Value*	[95% CI]	HR	*p Value*	[95% CI]
Age	0.91	0.000	0.88–0.94	0.9	0.000	0.87–0.93
Gender (male)	0.78	0.14	0.56–1.08	0.71	0.05	0.51–1.0
aHUS	1.76	0.14	0.82–3.76	1.92	0.09	0.9–4.13
STEC-HUS*	-	-	-	-	-	-
Transplantation	0.64	0.000	0.59–0.69	0.63	0.000	0.58–0.67
Haemodialysis	0.96	0.27	0.9–1.02	0.98	0.75	0.92–1.05
Peritoneal dialysis	1.07	0.02	1.01–1.15	1.07	0.041	1.002–1.14

*STEC-HUS lack of deaths in follow-up, *HR* hazard ratio, *CI* confidence interval

**Table 3:** Causes of death in children on KRT; aHUS CKD5 group and non-HUS CKD5 group

Cause of death	non-HUS* (*n* = 107)	aHUS (*n* = 7)	*p Value*
**Cardiovascular**	**43 (40%)**	**4 (57%)**	**0.02**
Cardiac arrest/sudden death	14	1	
Other causes of cardiac failure	14	1	
Myocardial ischemia/infarction	0	1	
Cerebrovascular accident	8	1	
Other cardiovascular	7	0	
**Infections**	**27 (25%)**	**1 (14%)**	**0.36**
Septicaemia	18	0	
Other infections	9	1	
**Malignancy**	**9 (8%)**	**0**	**0.58**
**Other identified causes**	**28 (26%)**	**2 (29%)**	**0.6**

*Cause of death provided for 107 of 140 deceased individuals in the non-HUS group

## Discussion

The work presented here is, to our knowledge, the first prospective cohort study based on a national KRT registry to analyse the fate of children with CKD5 in the course of HUS, after the introduction of eculizumab for aHUS and volume expansion treatment for STEC-HUS. Patients were classified as STEC-HUS or aHUS according to the registry’s codification of primary kidney disease, but in some unclear cases, the diagnosis could be changed retrospectively according to the course of the disease. Both aHUS and STEC-HUS are recognised as rare diseases in children. The incidence for STEC-HUS is relatively constant in the paediatric population in Europe, averaging 5.6/marp/year, with 1.4–4% (0.08–0.22/marp/year) of children developing CKD5 [7, 14, 26]. In the study group, the incidence for CKD5 in the course of STEC-HUS at 24-year follow-up has decreased, with a mean of 0.09/marp, which, when converted to the mean incidence in Europe, indicates a complete loss of kidney function in 1.6% of patients after STEC-HUS. For aHUS, according to Noris, the mean annual incidence is 0.5–2/marp, and in a population-based study in Italy, according to Ardissino, it is 0.75/marp, of which 30–50% of patients in the pre-eculizumab era developed CKD5. In Poland, between 2000 and 2023, 39 children with aHUS were on maintenance KRT, corresponding to an incidence of CKD5 due to aHUS of 0.19/marp. Assuming a similar incidence for the Polish paediatric population as in Italy, as Polish epidemiological data are not published, it can be concluded that 25.3% of children diagnosed with aHUS developed CKD5. At the same time, it should be emphasised that the incidence has decreased; as of 2018, no new cases of CKD5 have been reported in Poland in the course of STEC-HUS and as of 2020 in the course of aHUS (Fig 2).

It should be noted that STEC-HUS is an infectious disease that can be endemic, and unpublished epidemiological data on the incidence of STEC-HUS suggest that the incidence has remained constant or increased over the past 10 years, but this has not translated into the number of new CKD5 patients with this diagnosis in our registry. Diagnostic tools to confirm STEC infection have changed over the years. Currently available rapid microbiological methods could contribute to appropriate early patient management and subsequently improved outcomes. So the likely reason for better outcome is a change in the management of patients in the acute phase of the disease taking into account rapid volume expansion to avoid haemoconcentration as a risk factor for organ damage and poor long-term outcome [18, 20, 27]. There is no national protocol for symptomatic treatment in the acute phase of the disease in Poland, but the introduction of intensive fluid management in STEC-HUS, even for oligoanuric children, proposed by Ardissino in 2016, could have influenced the course of the disease. Unfortunately, information on treatment in the acute phase of HUS has not been reported to the KRT registry, so we cannot rule out the possibility of variability in protocols between centres. However, the improved outcome observed in recent years is likely due to the introduction of the new treatment protocols. The observed reduction in progression to CKD5 following STEC-HUS in childhood is also due to the widespread use of antihypertensive and nephroprotective therapy, but there is no single valid national protocol, so results may vary between centres.

Long-term follow-up is needed, including after the age of 18, as only in rare cases loss of kidney function occurs soon after an episode of AKI in STEC-HUS. In the case of aHUS, the lack of new patients starting KRT for this reason after 2020 is related to the availability of eculizumab since 2018 in Poland, which, if given in the first days after onset, is very effective in inhibiting the coagulation cascade and minimising the risk of irreversible kidney damage [28–30]. In exceptional situations, when the diagnosis is made too late and the drug is administered late, or in cases of treatment failure when the disease is not associated with complement activation, such as in patients with *DGKE* or *MMACHC* mutations, CKD5 may develop despite the use of eculizumab [31].

Discontinuation of C5 inhibitor for selected patients is safe and has been confirmed in many observations; however, patients with a known high risk of relapse are also at risk for irreversible kidney damage in the future [32, 33]. There have been no new cases of complete loss of kidney function during the period of eculizumab availability, despite the observed steady number of new cases of aHUS, making it foreseeable that it is likely that chronic KRT for this reason will only be given incidentally in future years.

The rapid development of genetic research observed in the last decade has contributed to the identification of congenital causes of HUS and the understanding of its pathogenesis, although the underlying genetic abnormality is still not found in about 40% of cases [22, 24, 32]. In the majority of cases (50–60%), aHUS is associated with pathogenic genetic variants causing complement system dysfunction or with the presence of acquired antibodies against Factor H of the complement system [31, 32, 34, 35]. The course of the disease largely depends on its genetic background. In our study population, we checked the familial burden for kidney disease, finding only one case of aHUS in three family members with the same diagnosis. A limitation of the study is the lack of information on genetic analyses of children with aHUS, but it should be noted that most patients started treatment when knowledge of potential pathogenic variants and the availability of genetic testing was limited. In the current state of knowledge, it seems clear that all patients with CKD5 in the course of aHUS, and also their family members in selected cases, should have an up-to-date genetic diagnosis performed [28]. Due to the high risk of aHUS recurrence after kidney transplantation and graft loss, transplants from living donors, especially related donors, have so far been avoided in this group of patients [12]. The exclusion of pathogenic variants in a related potential kidney donor is essential, as aHUS can manifest at any age, even in hitherto completely healthy adults. For this reason, in the study group, out of 40 kidney transplants performed, only in 2 cases were the donors family members. Nowadays, with the availability of eculizumab, which is recognised as a first-line drug both for the first episode of aHUS and relapses, as well as for prophylaxis after kidney transplantation, the procedure of transplantation from living donors, including related donors, after genetic testing in the donor and recipient and risk assessment, seems safer [36]. It should be noted that still approximately 60% of patients in the Polish population do not have currently known mutations causing aHUS, which needs to be re-evaluated in the future with new possibilities for genetic analyses (unpublished data).

Hypertension, which is significantly more common in children with HUS, is a recognised modifiable factor in the progression of CKD and a risk factor for organ complications, including ischaemic neurological episodes [37–39]. The progression of CKD is also influenced by underlying disease activity or recurrence. A significantly slower progression of CKD from AKI to CKD5 was observed in children with STEC-HUS compared with aHUS. The majority of aHUS patients started maintenance KRT within 1 year of the first episode of the disease. It should be noted that 8 patients out of 39 (20.5%) in the aHUS group had complete loss of kidney function and required maintenance KRT at the time of the first manifestation of the disease. A second factor contributing to the observed faster progression of CKD in aHUS patients may have been relapses of the underlying disease, but information on relapses was not recorded in the registry, which is a limitation of the study.

In some cases, despite KRT as AKI treatment for more than 3 months, which according to the RIFLE classification entitles to a diagnosis of CKD5, kidney function may improve. According to Tang et al. kidney function of patients with HUS chronically treated with KRT improves significantly more often compared to CKD5 cases of other aetiology [17]. In the study population, kidney function improved enough to allow KRT to be discontinued in 2 children (11%) with STEC-HUS and 6 children (15%) with aHUS, significantly more often compared with non-HUS causes of CKD5 (2.5%, *p* < 0.001). During follow-up, until patients reached 18 years of age, 2/2 with STEC-HUS and 3/6 with aHUS were deemed not to require KRT. The remaining 3/6 with aHUS required re-initiation of KRT after 2.75 years (median). According to Alconcher et al. a 5- or 10-year follow-up of patients after STEC-HUS is insufficient to establish the absence of features of kidney damage, as after a > 15-year follow-up period 51% manifested kidney sequelae and 14% developed CKD 2–5 [15]. Hence, it seems that long-term follow-up and nephrological care are essential, even after improvement or normalisation of kidney function is observed. Regular monitoring of GFR is particularly important into adulthood, due to anthropometric and behavioural changes in patients, in order to implement appropriate treatment of modifiable risk factors, such as hypertension, proteinuria, and recurrent disease for aHUS, if symptoms of CKD are found. Despite nephroprotective treatment, it cannot be excluded that some patients will develop CKD5 in adulthood.

In the study population, both the cause of CKD and the type of KRT used affected patient survival. In the ANZDATA registry, no differences in survival were observed for patients with HUS versus other causes of CKD5, but a significant limitation of the study was that it did not take into account the division into aHUS and STEC-HUS [17]. In the study group, 1-, 5-, and 10-year overall survival was best (100%) for children with STEC-HUS diagnosis—no deaths were observed during follow-up. In the group of children with aHUS, survival was significantly worse compared to other causes of CKD (10-year survival 78% vs. 87%), confirming previous observations, prior to the era of eculizumab treatment [22, 24, 25]. The type of dialysis used had a statistically significant effect on survival, although children on PD had only a slightly worse prognosis. On the other hand, kidney transplantation was a factor that significantly improved prognosis, but in the group of patients with aHUS, it was also associated with significantly shorter kidney graft survival compared with the control group. Recurrence of underlying disease in the transplanted kidney is a known risk factor for kidney graft loss before the era of C5 complement inhibitor therapy [17, 36]. The recurrence rate was approximately 40%, and the recurrence rate in the first year after kidney transplantation was 50–90%, with an 80–90% risk of kidney graft loss [12, 24]. The risk of relapse depends on the type of mutation found and is highest for *CFH*, *CFI* and *C3* variants [12, 24, 36]. In the ANZDATA registry study, kidney graft survival 1 year after transplantation was 73% versus 91% in the other causes of CKD group, but a division between STEC-HUS and aHUS was not included. In the UK aHUS registry, 1-year kidney graft survival in the period before the availability of eculizumab was 64% and was associated with recurrent thrombotic microangiopathy in the transplanted kidney in 42.4% of patients—after the introduction of eculizumab, these were respectively 97% and 2.6%. The Polish registry did not include information on recurrence in the transplanted kidney, which is a limitation of the study, but 5-year kidney graft survival in aHUS was significantly worse than in STEC-HUS and controls, at 77% versus 93% and 90%, respectively. Cardiovascular causes remain the leading cause of death, irrespective of age, in patients undergoing KRT [40]. In the case of aHUS, the underlying thrombotic microangiopathy and the possible multi-organ localisation of the lesions are particularly predisposing to vascular complications. Patients diagnosed with CKD5 due to aHUS in the study group were significantly more likely to die of cardiovascular causes compared to the control group (57% vs. 40%). The immediate causes of death were sudden cardiac arrest, heart failure, myocardial ischaemia, or cerebrovascular incident. It now appears that due to the availability and efficacy of targeted treatment of CM-HUS, severe vascular complications in this patient group will only be observed incidentally.

## Conclusions

Haemolytic uremic syndrome is a devastating disease with high risk of permanent kidney damage following initial disease episode. In recent years, both CM-HUS and STEC-HUS have become increasingly rare causes of CKD5 in children. Since the introduction of anti-C5 blocking antibody therapy for the treatment of CM-HUS, no child has been reported to start KRT for this reason. The development of CKD5 in childhood as a late complication of STEC-HUS has declined dramatically, probably due to advances in symptomatic treatment of AKI and widespread use of nephroprotective therapy. Children requiring maintenance KRT following STEC-HUS had a significantly better long-term survival, a shorter waiting time for kidney transplantation and better graft survival compared with children with CM-HUS and those with other causes of CKD5. In the pre-eculizumab era, children on KRT following complement-mediated HUS also had significantly worse survival rates, longer waiting time until kidney transplantation, and significantly worse graft survival compared with children with non-HUS causes of CKD5.

## Supplementary Information

Below is the link to the electronic supplementary material.

Graphical abstract (PPTX 117 KB)

## Data Availability

Restrictions apply to the availability of these data. Data were obtained from the Polish Society of Pediatric Nephrology and are available from the authors with the permission of the Polish Society of Pediatric Nephrology.

## References

[1] Bruyand M, Mariani-Kurkdjian P, Gouali M, de Valk H, King LA, Le Hello S, Bonacorsi S, Loirat C (2018) Hemolytic uremic syndrome due to Shiga toxin-producing Escherichia coli infection. Med Mal Infect 48:167–174. 10.1016/j.medmal.2017.09.012 29054297 10.1016/j.medmal.2017.09.012

[2] Tanné C, Javouhey E, Boyer O, Recher M, Allain-Launay E, Monet-Didailler C, Rouset-Rouvière C, Ryckewaert A, Nobili F, Gindre FA, Rambaud J, Duncan A, Berthiller J, Bacchetta J, Sellier-Leclerc AL (2022) Cardiac involvement in pediatric hemolytic uremic syndrome. Pediatr Nephrol 37:3215–3221. 10.1007/s00467-022-05427-2 35286451 10.1007/s00467-022-05427-2

[3] Costigan C, Raftery T, Carroll AG, Wildes D, Reynolds C, Cunney R, Dolan N, Drew RJ, Lynch BJ, O’Rourke DJ, Stack M, Sweeney C, Shahwan A, Twomey E, Waldron M, Riordan M, Awan A, Gorman KM (2022) Neurological involvement in children with hemolytic uremic syndrome. Eur J Pediatr 181:501–512. 10.1007/s00431-021-04200-1 34378062 10.1007/s00431-021-04200-1 PMC8821508

[4] Schaefer F, Ardissino G, Ariceta G, Fakhouri F, Scully M, Isbel N, Lommelé Å, Kupelian V, Gasteyger C, Greenbaum LA, Johnson S, Ogawa M, Licht C, Vande Walle J, Frémeaux-Bacchi V (2018) Clinical and genetic predictors of atypical hemolytic uremic syndrome phenotype and outcome. Kidney Int 94:408–418. 10.1016/j.kint.2018.02.029 29907460 10.1016/j.kint.2018.02.029

[5] Scheiring J, Andreoli SP, Zimmerhackl LB (2008) Treatment and outcome of Shiga-toxin-associated hemolytic uremic syndrome (HUS). Pediatr Nephrol 23:1749–1760. 10.1007/s00467-008-0935-6 18704506 10.1007/s00467-008-0935-6 PMC6901419

[6] Noris M, Remuzzi G (2009) Atypical hemolytic-uremic syndrome. N Engl J Med 361:1676–1687 19846853 10.1056/NEJMra0902814

[7] Ardissino G, Salardi S, Colombo E, Testa S, Borsa-Ghiringhelli N, Paglialonga F, Paracchini V, Tel F, Possenti I, Belingheri M, Civitillo CF, Sardini S, Ceruti R, Baldioli C, Tommasi P, Parola L, Russo F, Tedeschi S (2016) Epidemiology of haemolytic uremic syndrome in children. Data from the North Italian HUS network. Eur J Pediatr 175:465–473. 10.1007/s00431-015-2642-1 26498648 10.1007/s00431-015-2642-1

[8] Ashida A, Matsumura H, Shimono A, Fujii Y, Yamazaki S (2023) Comparison of outcomes after plasma therapy or eculizumab in pediatric patients with atypical hemolytic uremic syndrome. Clin Exp Nephrol 27:161–170. 10.1007/s10157-022-02293-y 36336723 10.1007/s10157-022-02293-y

[9] Kato H, Nangaku M, Hataya H, Sawai T, Ashida A, Fujimaru R, Hidaka Y, Kaname S, Maruyama S, Yasuda T, Yoshida Y, Ito S, Hattori M, Miyakawa Y, Fujimura Y, Okada H, Kagami S (2016) Clinical guides for atypical hemolytic uremic syndrome in Japan. Pediatr Int 58:549–555. 10.1111/ped.13044 27460397 10.1111/ped.13044

[10] Medina PJ, Sipols JM, George JN (2001) Drug-associated thrombotic thrombocytopenic purpura-hemolytic uremic syndrome. Curr Opin Hematol 8:286–293. 10.1097/00062752-200109000-00004 11604563 10.1097/00062752-200109000-00004

[11] Ong KL, Apostal M, Comstock N, Hurd S, Webb TH, Mickelson S, Scheftel J, Smith G, Shiferaw B, Boothe E, Gould LH (2012) Strategies for surveillance of pediatric hemolytic uremic syndrome: Foodborne Diseases Active Surveillance Network (FoodNet), 2000–2007. Clin Infect Dis 54(Suppl 5):S424-431. 10.1093/cid/cis208 22572665 10.1093/cid/cis208 PMC3348948

[12] Loirat C, Frémeaux-Bacchi V (2011) Atypical hemolytic uremic syndrome. Orphanet J Rare Dis 6:60. 10.1186/1750-1172-6-60 21902819 10.1186/1750-1172-6-60 PMC3198674

[13] Alfandary H, Rinat C, Gurevich E, Eisenstein I, Goldberg O, Kropach N, Landau D (2020) Hemolytic uremic syndrome: a contemporary pediatric experience. Nephron 144:109–117. 10.1159/000505401 31935726 10.1159/000505401

[14] Rosales A, Hofer J, Zimmerhackl LB, Jungraithmayr TC, Riedl M, Giner T, Strasak A, Orth-Höller D, Würzner R, Karch H (2012) Need for long-term follow-up in enterohemorrhagic Escherichia coli-associated hemolytic uremic syndrome due to late-emerging sequelae. Clin Infect Dis 54:1413–1421. 10.1093/cid/cis196 22412065 10.1093/cid/cis196

[15] Alconcher LF, Lucarelli LI, Bronfen S, Villarreal F (2024) Kidney sequelae in 281 Shiga toxin-producing Escherichia coli-hemolytic uremic syndrome patients after a median follow-up of 12 years. Pediatr Nephrol 39:1221–1228. 10.1007/s00467-023-06183-7 37880381 10.1007/s00467-023-06183-7

[16] Mody RK, Gu W, Griffin PM, Jones TF, Rounds J, Shiferaw B, Tobin-D’Angelo M, Smith G, Spina N, Hurd S, Lathrop S, Palmer A, Boothe E, Luna-Gierke RE, Hoekstra RM (2015) Postdiarrheal hemolytic uremic syndrome in United States children: clinical spectrum and predictors of in-hospital death. J Pediatr 166:1022–1029. 10.1016/j.jpeds.2014.12.064 25661408 10.1016/j.jpeds.2014.12.064

[17] Tang W, Mohandas J, McDonald SP, Hawley CM, Badve SV, Boudville N, Brown FG, Clayton PA, Wiggins KJ, Bannister KM, Campbell SB, Johnson DW (2012) End-stage kidney disease due to haemolytic uraemic syndrome–outcomes in 241 consecutive ANZDATA registry cases. BMC Nephrol 13:164. 10.1186/1471-2369-13-164 23206870 10.1186/1471-2369-13-164 PMC3544575

[18] Ardissino G, Tel F, Possenti I, Testa S, Consonni D, Paglialonga F, Salardi S, Borsa-Ghiringhelli N, Salice P, Tedeschi S, Castorina P, Colombo RM, Arghittu M, Daprai L, Monzani A, Tozzoli R, Brigotti M, Torresani E (2016) Early volume expansion and outcomes of hemolytic uremic syndrome. Pediatrics 137(1):e20153524. 10.1542/peds.2015-2153 10.1542/peds.2015-2153 26644486

[19] Loos S, Oh J, van de Loo L, Kemper MJ, Blohm M, Schild R (2021) Hemoconcentration and predictors in Shiga toxin-producing E. coli-hemolytic uremic syndrome (STEC-HUS). Pediatr Nephrol 36:3777–3783. 10.1007/s00467-021-05108-6 34046736 10.1007/s00467-021-05108-6 PMC8497454

[20] Ake JA, Jelacic S, Ciol MA, Watkins SL, Murray KF, Christie DL, Klein EJ, Tarr PI (2005) Relative nephroprotection during Escherichia coli O157:H7 infections: association with intravenous volume expansion. Pediatrics 115:e673-680. 10.1542/peds.2004-2236 15930195 10.1542/peds.2004-2236

[21] Ardissino G, Daccò V, Testa S, Civitillo CF, Tel F, Possenti I, Belingheri M, Castorina P, Bolsa-Ghiringhelli N, Tedeschi S, Paglialonga F, Salardi S, Consonni D, Zoia E, Salice P, Chidini G (2015) Hemoconcentration: a major risk factor for neurological involvement in hemolytic uremic syndrome. Pediatr Nephrol 30:345–352. 10.1007/s00467-014-2918-0 25149851 10.1007/s00467-014-2918-0

[22] Noris M, Caprioli J, Bresin E, Mossali C, Pianetti G, Gamba S, Daina E, Fenili C, Castelletti F, Sorosina A, Piras R, Donadelli R, Maranta R, van der Meer I, Conway EM, Zipfel PF, Goodship TH, Remuzzi G (2010) Relative role of genetic complement abnormalities in sporadic and familial aHUS and their impact on clinical phenotype. Clin J Am Soc Nephrol 5:1844–1859. 10.2215/CJN.02210310 20595690 10.2215/CJN.02210310 PMC2974386

[23] Caprioli J, Noris M, Brioschi S, Pianetti G, Castelletti F, Bettinaglio P, Mele C, Bresin E, Cassis L, Gamba S, Porrati F, Bucchioni S, Monteferrante G, Fang CJ, Liszewski MK, Kavanagh D, Atkinson JP, Remuzzi G (2006) Genetics of HUS: the impact of MCP, CFH, and IF mutations on clinical presentation, response to treatment, and outcome. Blood 108:1267–1279. 10.1182/blood-2005-10-007252 16621965 10.1182/blood-2005-10-007252 PMC1895874

[24] Fremeaux-Bacchi V, Fakhouri F, Garnier A, Bienaimé F, Dragon-Durey MA, Ngo S, Moulin B, Servais A, Provot F, Rostaing L, Burtey S, Niaudet P, Deschênes G, Lebranchu Y, Zuber J, Loirat C (2013) Genetics and outcome of atypical hemolytic uremic syndrome: a nationwide French series comparing children and adults. Clin J Am Soc Nephrol 8:554–562. 10.2215/CJN.04760512 23307876 10.2215/CJN.04760512 PMC3613948

[25] Halimi JM, Al-Dakkak I, Anokhina K, Ardissino G, Licht C, Lim WH, Massart A, Schaefer F, Walle JV, Rondeau E (2023) Clinical characteristics and outcomes of a patient population with atypical hemolytic uremic syndrome and malignant hypertension: analysis from the Global aHUS registry. J Nephrol 36:817–828. 10.1007/s40620-022-01465-z 36152218 10.1007/s40620-022-01465-z PMC10090001

[26] Alconcher LF, Lucarelli LI, Bronfen S (2023) Long-term kidney outcomes in non-dialyzed children with Shiga-toxin Escherichia coli associated hemolytic uremic syndrome. Pediatr Nephrol 38:2131–2136. 10.1007/s00467-022-05851-4 36595068 10.1007/s00467-022-05851-4

[27] Ardissino G, Tel F, Testa S, Paglialonga F, Longhi S, Martelli L, Consolo S, Picicco D, Dodaro A, Daprai L, Colombo R, Arghittu M, Perrone M, Chidini G, Scalia Catenacci S, Cropanese I, Consonni D (2018) A simple prognostic index for Shigatoxin-related hemolytic uremic syndrome at onset: data from the ItalKid-HUS network. Eur J Pediatr 177:1667–1674. 10.1007/s00431-018-3198-7 30094644 10.1007/s00431-018-3198-7

[28] Loirat C, Fakhouri F, Ariceta G, Besbas N, Bitzan M, Bjerre A, Coppo R, Emma F, Johnson S, Karpman D, Landau D, Langman CB, Lapeyraque AL, Licht C, Nester C, Pecoraro C, Riedl M, van de Kar NC, Van de Walle J, Vivarelli M, Frémeaux-Bacchi V (2016) An international consensus approach to the management of atypical hemolytic uremic syndrome in children. Pediatr Nephrol 31:15–39. 10.1007/s00467-015-3076-8 25859752 10.1007/s00467-015-3076-8

[29] Legendre CM, Licht C, Muus P, Greenbaum LA, Babu S, Bedrosian C, Bingham C, Cohen DJ, Delmas Y, Douglas K, Eitner F, Feldkamp T, Fouque D, Furman RR, Gaber O, Herthelius M, Hourmant M, Karpman D, Lebranchu Y, Mariat C, Menne J, Moulin B, Nürnberger J, Ogawa M, Remuzzi G, Richard T, Sberro-Soussan R, Severino B, Sheerin NS, Trivelli A, Zimmerhackl LB, Goodship T, Loirat C (2013) Terminal complement inhibitor eculizumab in atypical hemolytic-uremic syndrome. N Engl J Med 368:2169–2181. 10.1056/NEJMoa1208981 23738544 10.1056/NEJMoa1208981

[30] Walle JV, Delmas Y, Ardissino G, Wang J, Kincaid JF, Haller H (2017) Improved renal recovery in patients with atypical hemolytic uremic syndrome following rapid initiation of eculizumab treatment. J Nephrol 30:127–134. 10.1007/s40620-016-0288-3 26995002 10.1007/s40620-016-0288-3 PMC5316393

[31] Brocklebank V, Walsh PR, Smith-Jackson K, Hallam TM, Marchbank KJ, Wilson V, Bigirumurame T, Dutt T, Montgomery EK, Malina M, Wong EKS, Johnson S, Sheerin NS, Kavanagh D (2023) Atypical hemolytic uremic syndrome in the era of terminal complement inhibition: an observational cohort study. Blood 142:1371–1386. 10.1182/blood.2022018833 37369098 10.1182/blood.2022018833 PMC10651868

[32] Wijnsma KL, Duineveld C, Wetzels JFM, van de Kar N (2019) Eculizumab in atypical hemolytic uremic syndrome: strategies toward restrictive use. Pediatr Nephrol 34:2261–2277. 10.1007/s00467-018-4091-3 30402748 10.1007/s00467-018-4091-3 PMC6794245

[33] Fakhouri F, Zuber J, Frémeaux-Bacchi V, Loirat C (2017) Haemolytic uraemic syndrome. Lancet 390:681–696. 10.1016/S0140-6736(17)30062-4 28242109 10.1016/S0140-6736(17)30062-4

[34] Goodship TH, Cook HT, Fakhouri F, Fervenza FC, Frémeaux-Bacchi V, Kavanagh D, Nester CM, Noris M, Pickering MC, Rodríguez de Córdoba S, Roumenina LT, Sethi S, Smith RJ (2017) Atypical hemolytic uremic syndrome and C3 glomerulopathy: conclusions from a “Kidney Disease: Improving Global Outcomes” (KDIGO) Controversies Conference. Kidney Int 91:539–551. 10.1016/j.kint.2016.10.005 27989322 10.1016/j.kint.2016.10.005

[35] Lemaire M, Noone D, Lapeyraque AL, Licht C, Frémeaux-Bacchi V (2021) Inherited kidney complement diseases. Clin J Am Soc Nephrol 16:942–956. 10.2215/CJN.11830720 33536243 10.2215/CJN.11830720 PMC8216622

[36] Glover EK, Smith-Jackson K, Brocklebank V, Wilson V, Walsh PR, Montgomery EK, Wong EKS, Johnson S, Malina M, Kavanagh D, Sheerin NS (2023) Assessing the impact of prophylactic eculizumab on renal graft survival in atypical hemolytic uremic syndrome. Transplantation 107:994–1003. 10.1097/TP.0000000000004355 36413152 10.1097/TP.0000000000004355 PMC10065821

[37] Sanders E, Brown CC, Blaszak RT, Crawford B, Prodhan P (2021) Cardiac manifestation among children with hemolytic uremic syndrome. J Pediatr 235:144-148.e144. 10.1016/j.jpeds.2021.03.067 33819463 10.1016/j.jpeds.2021.03.067 PMC8316308

[38] Loirat C, Macher MA, Elmaleh-Berges M, Kwon T, Deschênes G, Goodship TH, Majoie C, Davin JC, Blanc R, Savatovsky J, Moret J, Fremeaux-Bacchi V (2010) Non-atheromatous arterial stenoses in atypical haemolytic uraemic syndrome associated with complement dysregulation. Nephrol Dial Transplant 25:3421–3425. 10.1093/ndt/gfq319 20530807 10.1093/ndt/gfq319

[39] Davin JC, Majoie C, Groothoff J, Gracchi V, Bouts A, Goodship TH, Loirat C (2011) Prevention of large-vessel stenoses in atypical hemolytic uremic syndrome associated with complement dysregulation. Pediatr Nephrol 26:155–157. 10.1007/s00467-010-1608-9 20652819 10.1007/s00467-010-1608-9 PMC2991236

[40] Shroff R, Ledermann S (2009) Long-term outcome of chronic dialysis in children. Pediatr Nephrol 24:463–474. 10.1007/s00467-007-0700-2 18214549 10.1007/s00467-007-0700-2 PMC2755764

