# Supplementary material for: Haemolytic uremic syndrome as a cause of chronic kidney disease stage 5 in children is in retreat: results from the Polish Registry of Kidney Replacement Therapy in children (2000–2023)
Source: Pediatr Nephrol. 2024 Nov 16;40(4):1069–79. doi: 10.1007/s00467-024-06584-2 (PMC11885394; doi:10.1007/s00467-024-06584-2)
Supplement: Supplementary file 1 — Graphical abstract (PPTX 117 KB) [file 467_2024_6584_MOESM1_ESM.pptx]

## Slide 1
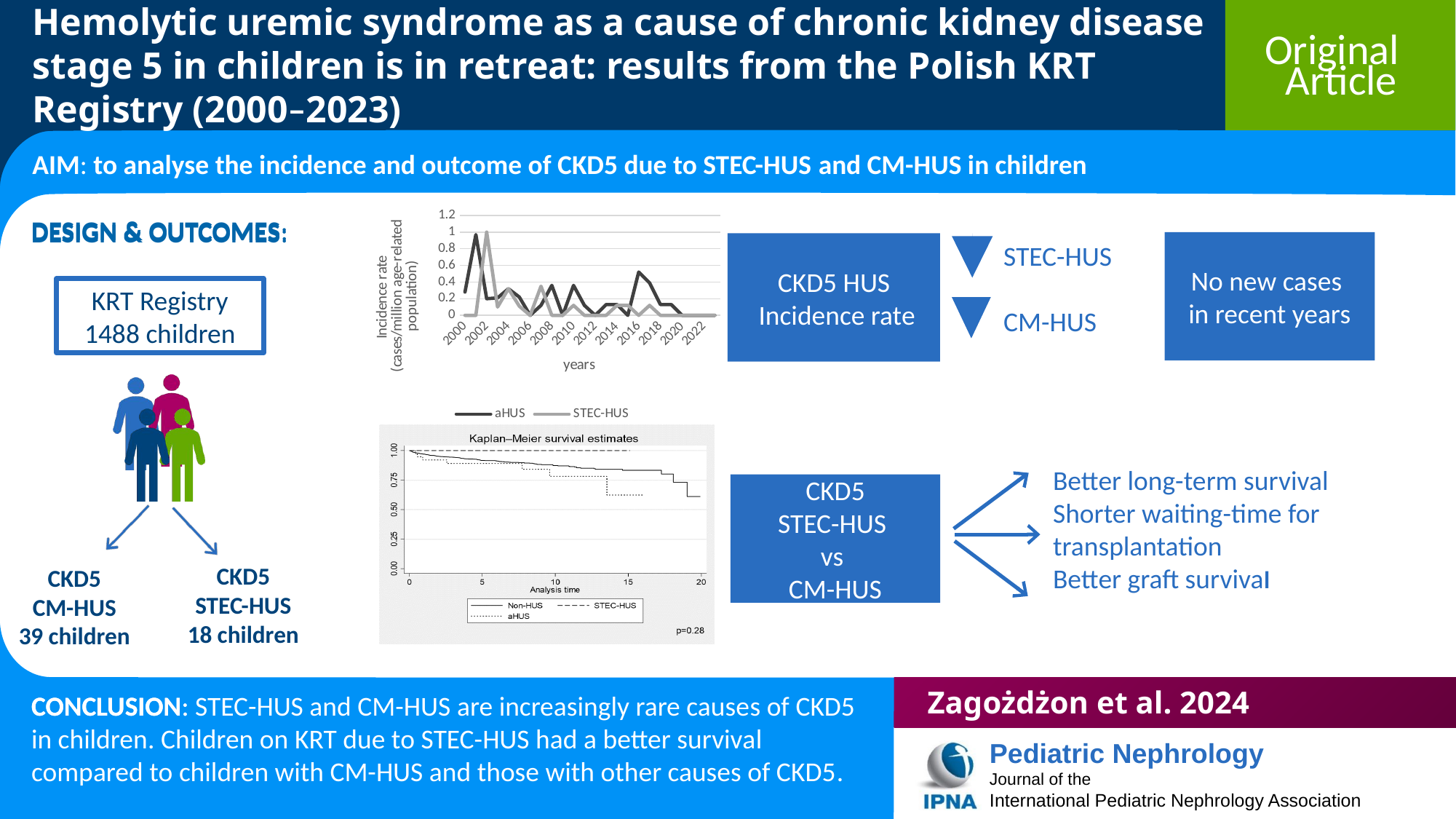

Hemolytic uremic syndrome as a cause of chronic kidney disease stage 5 in children is in retreat: results from the Polish KRT Registry (2000–2023)
AIM: to analyse the incidence and outcome of CKD5 due to STEC-HUS and CM-HUS in children
### Chart
| Category | aHUS | STEC-HUS |
|---|---|---|
| 2000 | 0.28 | 0.0 |
| 2001 | 0.97 | 0.0 |
| 2002 | 0.2 | 1.0 |
| 2003 | 0.21 | 0.1 |
| 2004 | 0.32 | 0.32 |
| 2005 | 0.22 | 0.11 |
| 2006 | 0.0 | 0.0 |
| 2007 | 0.12 | 0.35 |
| 2008 | 0.36 | 0.0 |
| 2009 | 0.0 | 0.0 |
| 2010 | 0.36 | 0.12 |
| 2011 | 0.12 | 0.0 |
| 2012 | 0.0 | 0.0 |
| 2013 | 0.13 | 0.0 |
| 2014 | 0.13 | 0.12 |
| 2015 | 0.0 | 0.12 |
| 2016 | 0.52 | 0.0 |
| 2017 | 0.39 | 0.12 |
| 2018 | 0.13 | 0.0 |
| 2019 | 0.13 | 0.0 |
| 2020 | 0.0 | 0.0 |
| 2021 | 0.0 | 0.0 |
| 2022 | 0.0 | 0.0 |
| 2023 | 0.0 | 0.0 |DESIGN & OUTCOMES:
DESIGN & OUTCOMES:
No new cases
in recent years
STEC-HUS
CM-HUS
CKD5 HUS
 Incidence rate
KRT Registry
1488 children
Better long-term survival
Shorter waiting-time for transplantation
Better graft survival
CKD5
STEC-HUS
vs
CM-HUS
CKD5
STEC-HUS
18 children
CKD5
CM-HUS
39 children
Zagożdżon et al. 2024
CONCLUSION:
CONCLUSION: STEC-HUS and CM-HUS are increasingly rare causes of CKD5 in children. Children on KRT due to STEC-HUS had a better survival compared to children with CM-HUS and those with other causes of CKD5.
